# The invisible hands in policy making: A qualitative study of the role of advocacy in priority setting for maternal and child health in Nigeria

**DOI:** 10.34172/hpp.2023.18

**Published:** 2023-07-10

**Authors:** Chinyere Okeke, Benjamin Uzochukwu, Maylene Shung-king, Lucy Gilson

**Affiliations:** ^1^Department of Community Medicine, College of Medicine, University of Nigeria Enugu-Campus, Enugu, Nigeria; ^2^Health Policy Research Group, University of Nigeria Enugu-Campus, Enugu Nigeria; ^3^Health Policy and Systems Division, School of Public Health and Family Medicine, University of Cape Town, Cape Town, South Africa

**Keywords:** Advocacy, Policy, Maternal-child health, Nigeria

## Abstract

**Background:**

Maternal and child health is a priority for most governments, especially those in low and middle-income countries (LMICs), due to high mortality rates. The combination of individual and social actions designed to gain political commitment, policy support and social acceptance for health goals are influenced by the interplay between the advocates and the strategies they deploy in planning and advocating for maternal and child health issue. This study aims to deepen our understanding of how advocacy has influenced maternal and child health priority setting in Nigeria.

**Methods:**

This is a mixed method study that involved 24 key informant interviews, document review, policy tracking and mapping of advocacy events that contributed to the repositioning of maternal and child health on the political agenda was done. Respondents were deliberately selected according to their roles and positions. Analysis was based on Shiffman and Smith’s policy analysis framework of agenda setting.

**Results:**

Our findings suggest that use of various strategies for advocacy such as influencers, media, generated different outcomes and the use of a combination of strategies was found to be more effective. The role of advocacy in issue emergence was prominent and the presence of powerful actors, favorable policy window helped achieve desired outcomes. The power of the advocates and the strength of the individuals involved played a key role.

**Conclusion:**

This study finds it possible to understand the role of advocacy in policy agenda setting through the application of agenda setting framework. To achieve the health SDG goals, advocacy barriers need to be addressed at multiple levels.

## Introduction

 Maternal and child health (MCH) has been a problem addressed by most governments, especially those in low and middle-income countries (LMICs), and for donors and non-governmental agencies, for several decades.^1^ This is particularly due to the high mortality rates recorded in these countries. Considerable progress has been made in reducing maternal, newborn, and child mortality worldwide, but only slow progress has been made in LMICs and many more deaths could be prevented if effective interventions were available to all who could benefit from them.^2,3^ In Nigeria, there has been fluctuating levels of prioritization of MCH among government policy-makers since the mid-2000s, with the introduction and cessation of many policies, programs and interventions. This has been said to have partly contributed to the slow progress made in tackling MCH mortality.^4^ The problem of sustaining political and bureaucratic commitment for the implementation and evolution of policies is a widely recognized problem.^5^ Waning commitment can lead to stagnation in implementation, as it undermines the likelihood that political and bureaucratic actors sustain such policies and strategies over time. There is therefore a need to maintain strategic issues on the political agenda by supporting their prioritization.

 Studies have shown that many factors influence whether issues evolve and stay on the national political agenda, such as the severity and group of people affected by the problem, the global and national attention given to the problem and presence of altruistically motivated individuals who initiate a campaign and lobby to draw awareness to an issue.^6-8^ Researchers have, however, recommended further, focused research on national-level priority setting in LMICs to deepen our understanding.

 Within the agenda-setting process, policy advocates can play an important role in portraying policy issues to gain political attention and support.^6^ They may include civil society organizations (CSOs), media, medical associations, academia etc. (who are they?) and they commonly act on how to move citizens’ voice from access, to presence, to influence,^9^ just as Moran et al refer to policy making as a process of ‘persuasion’.^10^ The advocates seek to revise and shape the policy agenda to enable its prioritization and ascendance into the political cycle.^11,12^ Some achieve this using credible, analyzed and simply-presented evidence, thereby bridging the gap between the evidence- production system and the policy community.^13^ By breaking the existing information silo, they connect the various relevant stakeholders to support policy change - as policy-makers are confronted with a multitude of competing issues and have limited resources for dealing with them.^14^ The effectiveness of advocacy has been documented and found to be influential in garnering political attention for a particular policy and in the introduction of many health interventions.^15,16^ Global attention on *maternal, *newborn and *child health* (MNCH) has, more specifically, been attributed to the result of advocacy by women’s rights activists. Studies have shown the effectiveness of advocacy in various settings,^17^ but there are few studies considering advocacy for MCH in Nigeria^18^ or other LMICs, considering the fact that the country is struggling to achieve set sustainable development goals. This paper aims, therefore, to examine how different advocacy and lobbying efforts influenced the (re)emergence of MNCH as a political priority in the Nigerian context in 2015. It draws on empirical data from a case study of maternal health policy and program evolution in 2015 following the withdrawal of a previous program (SURE-P MCH).

 This study adopts the World Health Organization (WHO) definition of advocacy for health, which states that it is a ‘combination of individual and social actions designed to gain political commitment, policy support, social acceptance and systems support for a particular health goal or programme’.^19^ This definition is in line with Tsui and colleagues’ definition of policy advocacy, more broadly, as “an intervention intended to catalyze, stimulate or otherwise seed some form of change through different forms of persuasion”^20^ as well as the widely known ‘Advocacy coalition framework’.^21^ Influencing public policy change is a difficult and complex task because policy change is itself a complex process, influenced by many actors and factors – ideology, values, power dynamics, available resources, interests, habits and traditions.^22^

 It has implications for government and non-governmental advocates aiming to sustain commitment to existing policies in changing political cycles and in the presence of a multitude of competing issues.

## Material and Methods

###  Study design

 This study was undertaken at the national and State levels. The Federal Capital Territory (FCT) Abuja was chosen because it is the capital of Nigeria and the country’s administrative and political centre. Anambra state was randomly chosen to explore the play out of events at the subnational level.

 This study adopted a qualitative study approach^23^ of discourse analysis type. It was conducted between June and December 2018, to answer the research question: How has advocacy influenced MCH priority setting following the withdrawal of SURE-P MCH program in 2015 in Nigeria? The phenomenon of inquiry is the role of advocacy in maintaining MNCH as a political priority on the political agenda. Qualitative approach was chosen because this study seeks to explore, explain and give understanding to a situation.^24^ Consolidated criteria for Reporting Qualitative Research has been applied here.^25^

###  Conceptual framework

 It uses the Shiffman and Smith framework,^26^ developed for assessing the factors that prioritize some issues over others in the process of agenda setting. Four elements describe what factors affect national agenda setting: the interaction between actors and their power, ideas, context of the political environment and characteristics of the issues themselves. This framework is said to be the most developed and comprehensive-related health policy framework.^27^ Also Shiffman in 2007 had explored how maternal health had gained priority and suggested that issues were said to have gained government attention when the national actors actively give attention to that issue; policies addressing the issue are enacted through an authoritative decision-making process and resources commensurate with the severity of the problem are provided.^17^ These will be used to assess the case of advocacy for MCH in Nigerian context.

 Smith and Shiffman’s framework states that political priority is seen when: political leaders publicly and privately express sustained concern for the issue; enactment of policies and laws by organisations and political systems to address the problem; and their provision of resources to the problem that are commensurate with its severity.^26^ These criterion were used here to show the outcomes of the advocacy events. The framework was selected for this analysis as it is a widely used and comprehensive framework (reference) Walt and Gilson. In this study the framework was used to analyse the data drawn from the Nigerian experience, and to consider whether additional factors were relevant in this experience.

 Smith and Shiffman’s framework was used to consider range of the factors that influence the agenda setting stage of the policy process.^26^ This framework (2007)^27^ identifies four determinants of political priority: the power of actors, the power of ideas, political contexts, and characteristics of the issues. This will help x-ray MNCH advocacy efforts in Nigeria, towards the effort to attain health SDG goals. This is shown in Table 1.^26^

**Table 1:** The four categories for the framework on determinants of political priority for initiatives

**Elements **	**Description **	**Factors shaping policy priorities **
Context	The environment in which actors operate	1. Policy windows: political moments when conditions align favourably for an issue, presenting opportunities for advocates to influence decision makers. 2. Global governance structure: the degree to which norms and institutions operating in a sector provide a platform for effective collective action.
Actor power	The strength of the individuals and networks concerned with the issue.	3. Policy community cohesion: the degree of coalescence in the network involved with the issue. 4. Leadership: the presence of individuals capable of uniting the policy community, acknowledged as strong champions. 5. Guiding institutions: effectiveness of organizations or coordinating mechanisms. 6. Civil society mobilization: the extent to which grassroots organizations are mobilized to support action.
Ideas	The ways in which those involved with the issue understand and portray it.	7. Internal frame: the degree to which the policy community agrees on the definition of, causes of and solutions to, the problem. 8. External frame: public portrayals of the issue in ways that resonate with external actors, especially the political leaders who control resources.
Issue characteristics	Features of the problem	9. Credible indicators: clear measures that show the severity of the problem, that can be used to monitor progress. 10. Severity: the size of the burden relative to others. 11.Effective interventions: the extent to which proposed means of addressing the problem are explained, cost effective, backed by scientific evidence, simple to implement and inexpensive.
**Outcomes: Political Priority** • When political leaders publicly and privately express sustained concern for the issue • When the organisations and political systems they lead enact policies to address the problem • When these organisations and political systems provide levels of resources to the problem that are commensurate with its severity.		

Source: Adapted from Shiffman and Smith.^25^

###  Data collection

####  Selection and description of participants

 Respondents were purposively selected from key stakeholders identified during the document review and advocacy events’ mapping done at the preliminary stage of this study, as advocates or policy makers involved in MCH in Nigeria, according to their role and position. The Table 2 below shows the profile of the participants.

**Table 2:** Socio-demographic profile of the participants

**Participants/Respondents codes**	**Location**	**Post**	**Total number**	**Male**	**Female**
Policy makers (national level) P1,2,3	FCT Abuja	Directors FMOH & Assistant	3	2	1
Policy maker subnational level (P4,5,6)	Anambra state	Chairman, State house of Assembly, Director & Assistant Commissioner	3	2	1
Development partners (D1,2,3)	Abuja	Director, UNICEF & UNDP	3	2	1
CSO (C1,2,3)	Abuja and Anambra	Head of Coalition group, Heads of CSO groups	3	1	2
NGO (N1,2,3)	Abuja and Anambra	Program officers	3	2	1
Professional groups (G1,2)	Anambra	Group leaders	2	1	1
Media (M1,2)	Abuja and Anambra	Health journalist	2	1	1
Academia/researcher (R1,2)	Abuja and Anambra	Professor and Lecturer	2	1	1
Community groups (G3)	Abuja	Heads of group	1		1
Advocacy Influencers (A1,2)	Abuja and Anambra		2	1	1

Table 2 shows the diverse participants recruited for the study, their positions and their sex.

 Data was collected using review of relevant documents and key informant interviews. Document review was conducted to inform the development of interview guide, identify the policies and programs carried out in maternal health in the past and to map out the activities of advocacy groups in MCH at the national and state levels. Landscape documents, national reports and policy analysis documents were reviewed. The document review mapped various advocacy and lobbying efforts in restructuring and repositioning MCH on the political agenda, post SURE-P. Information was collected with a proforma under the following headings: advocacy/ lobbying event and why; person /group that led the event; date and venue of the event; contextual features of the event; strategies used and the outcome of the event. Document review identified^28^ advocacy visits paid to the government at either the national or state level after the withdrawal of SURE-P program.

 Key in-depth interviews with participants were conducted face-to-face using a pre-tested semi-structured interview guide. The guide was pretested in Enugu State, and it explored: the context of MCH in Nigeria and how actors perceived maternal health as a problem; the strategies adopted by the actors, the outcome of the advocacy and what enabled or constrained the advocacy events. The findings from the pre-tests were used to review and refine the question guide prior to usage. Appointments were sought by cell phone and personal visits and all participants approached obliged. Participants were briefed on the purpose of the study, and they understood it prior to giving their written informed consent to be involved. Participants were interviewed alone in their offices. Interviews lasted about 60 minutes, were conducted in English, audiotaped with the consent of the respondent, transcribed verbatim and transcripts sent back to participants to confirm their credibility. Interviews were conducted by the principal investigator and a sociologist who served as a notetaker, and both are experienced qualitative researchers who conducted interviews until saturation was achieved. The participant-researcher relationship was a good one as diligence was applied. The researchers were also flexible, understanding, and respectful to the participants and their time. It was also a bit personal and friendly as both participants and researchers were highly interested in the subject and this led to the success of having all participants approached, grant interviews willingly.

###  Data analysis

 The document review was analysed using the manual content analysis method with a proforma. Information was extracted under the headings outlined in the proforma, following the headings identified from the Shiffman’s framework.^26^ Qualitative data audiotapes were transcribed verbatim, anonymised, double coded by the author and the note taker in MS Word using colour-coded highlights and analysed using manual thematic and framework analysis of the main topics outlined in the interview guide. Other codes not included in the guide emerged during the reading of the interviews. Inter-coder reliability was established by having a trained public health specialist code two transcripts to determine levels of consistency, and discrepancies were resolved through consensus building. Findings were supplemented and validated with document review. Also, a validation meeting was held, and participants mostly corroborated our findings and reiterated the need for more advocacy and coalitions to be built if MCH indices of Nigeria is to be improved.

## Results

 This is presented under the following headings: Perceived advocacy outcomes; Contexts that enabled advocacy to have effect, Power of the actors, the ideas and issue characteristics.

###  Perceived advocacy outcomes

####  Political leaders’ express of sustained concern for the issue

 Sustained concern for MNCH in the country by the policy makers was perceived to be a major outcome. All respondents attributed this to advocacy activities happening in the country which intensified following the withdrawal of SURE-P MCH program. One of the respondents said,

 “*Advocacy is very good... So, advocacy is a powerful tool because most of these people you know they are not health workers, the governor is not a medical doctor, so it is not like he doesn’t know, but when you come to him as an advocate and you are able to give him facts looking at indices and looking at what is on ground, telling him the gaps and everything, he will understand and he will quickly key into it, that is how we have been able to sustain things till now” *(A1).

####  Enactment of policies and laws by organisations and political systems to address the problem

 Some policies were perceived to be attributed to the advocacy activities that took place in those places. Free MNCH services at the state level was implemented as a response to the evidence presented by an advocacy group during one of their visits,

 “*If you have been to Bayelsa, you will know that a lot of their areas are surrounded by water. As rich as the government may be, the people living there were not having good medical care services. When we found out, what we did was to pay advocacy visits to the governor of Bayelsa and you don’t just go and sit in front of him and tell him he has poor indices, you will not score any point. What we do now is that even videos of how people were delivering there by putting herbs inside somebody’s body parts, by jumping on somebody’s tummy to push out the baby have been captured and how people died. These very graphic details were revealed to the governor, he said he never knew that it was as bad as that. We then signed a MOU with the state. The governor has bought deeply into it because in the state now, if you register for ante-natal, you are paid cash (CCT), if a man’s wife registers, same thing. There’s a policy to that effect” *(G1).

 Other policy issues perceived by respondents to be attributed to advocacy include Inclusion of MNCH interventions and packages in the current basic health care provision fund (BHCPF) scheme, which is a social health insurance scheme that will provide free care to mothers and children in the rural communities, especially for the indigents. A reporter for a media house had this to say,

 “*Yes, there are some advocacies that has been directed at MNCH issues that has been quite effective. One was, [apart from the larger national health care ACT], there has been consistent advocacy to get the BHCPF into the budget as done, but not just getting it into the budget but having it released and ear marked. That’s one of them and we know that the BHCPF mostly addresses free maternal and child health” *(M1).

 Also, the PHCUOR policy which has 6 main components and 3 of which relates to mothers and children. This was said to have been implemented in the sub-national levels through advocacy.

*“….in the passage of primary health care development agency bill in Anambra state, we championed it and paid advocacy visits to the house of assembly and the commissioner for health, then and the governor took it upon himself to send the bill as executive bill to the house of assembly. Following advocacy to the ministry of justice and other line ministries, it was passed and then we persevered until the board was inaugurated and members were appointed and inaugurated immediately, and they moved into action” *(C2).

####  Provision of resources to the problem that are commensurate with its severity

 This have been seen as a sign of political priority for a cause and was identified in this study,

 “*I remember one specifically, UNICEF is interested in maternal and child nutrition and when we developed the benefit package initially, we wanted to have a slim benefit package, so it had nothing on nutrition but there was this targeted advocacy to the Minister of Health and the Minister of Finance and eventually we agreed to add child nutrition to the benefit package” (D2).*

 Yet another respondent reiterated that increase in health budget and releases was due to their advocacy efforts,

*“…so I can say that the increase in the budget was as a result of that advocacy and the other advocacies that had happened in the past. So eventually, the 2018 budget for health was increased adequately.” (C3).*

 Interestingly, all respondents attested that material and human resources had been provided for MNCH due to advocacy, all these point to the fact that advocacy has had positive outcomes in Nigeria.

 “*We have seen cases whereby some line items have been removed from the budget or funding being cut, but because of our advocacy, we have been able to return those funding back and those funding received their appropriate attention, especially those for MNCH, even increased number of workforce and materials” (P6).*

####  Identified barriers to effective advocacy

 The identified barriers to effective advocacy include a) Lack of conceptualized, feasible, efficient and cost-effective solution to MNCH problems; b) Lack of coordination amongst the advocates as different groups were reported paying advocacy to the same politicians for the same issue at different times and the politicians found it to be quite irritating. Other barriers were found to be mostly contextual, such as the lack of job specification for the three tiers of government as the Nigerian constitution does not have a clearly defined role of specific health responsibility for the three tiers of government; Top-bottom approach to policy agenda setting and formulation with lack of input from the citizenry as stated by most of the respondents; Use of advocates who are not knowledgeable in that area and are not well equipped with the right information nor evidence; issues with trust relationship between the advocates and the government was more prominent at the subnational level, making it difficult to get commitments from the politicians.

 We look at what enabled advocacy to have effect, considering the framework and these include contexts, actor power, ideas and issue characteristics.

###  Contexts

####  Political context

 Participants attested to the fact that change in government at the moment helped the advocates pursue and convince the incoming government on the need to include MNCH on their campaign mandate, as this will attract the said population, mainly the women. After the elections, the politicians had to live to expectations and fulfil the promises they made when reminded by advocacy visits. Whereas respondents perceived that the politicians at the subnational level were not under social pressure and exhibited little political will, despite various advocacy visits. This was attributed to the fact that there was no change in government. Also, the government at this level was not eager to adopt the initiatives from the national since they were from opposition party.

####  Having a de-centralized health system

 Nigeria has a decentralized health system: the Federal, State and Local government. These are all responsible for the health of mothers and children, though the first point of call is the primary health centres which is the responsibility of the local government. This decentralized health systems has its merits and demerits. One of the national respondents said:

*“Now if you look at the Federal level, the problem is even more compounded because of the lopsided nature of the Nigerian government and the structure of Nigeria. Because this maternal and childcare issue is supposed to be a primary health issue, isn’t it? Now, this should be cascaded down to the States and Local Governments. But since there are Primary Health Centers that Federal Government is responsible for, tell me how they will be in Abuja taking care of the Primary Health Centre in my little village. That disconnection is part of the problem”. *(N3).

####  Having available policy/legal frameworks

 Most of the respondents stated that the existence of policies and legal framework for MCH in the country was an enabling ground for advocating and lobbying for MCH issues. The policy makers are more likely to agree to a cause that has a legal backing.

*“...having policy pronouncement and policy document as always is not the problem of Nigeria and Nigerian government but after getting these, implementing them becomes a problem because every government talks about the care for the mother and child, having adequate facilities, doing this and doing that. But, bringing that talk to work is a huge problem. We go with these policies, and they are more likely to agree.” (C3).*

###  Actor power

####  Make-up of the advocacy groups

 The advocates were similar at the national and sub national levels though more actors come to play at the national level, with a lot of international attention too. Some groups were homogenous, such as the professional groups with only doctors from one specialty advocating for mothers and children. Other times, the advocacy groups were composed of people from different backgrounds but with same vision and mission, pursuing same goal.

*“…advocacy is better when groups of people come together and have a common vision and through a coordinated activity, meet the right people and are given audience, then they are more likely to achieve their aims. *(G1).

####  Ability to attract resources for MNCH

 The MNCH advocacy groups were able to organize themselves, acquire resources, mobilize members some of whom are powerful and connected and can help the group in various ways. According to a respondent,

*“… so number one is the passion, coalitions are formed based on passion. Number two is the passion that drives the coalition. The third is the ability, the capacity of the coalition and then the unity of purpose. They must have a common vision to be able to achieve any result as a coalition. These were all present in our group”. *(C1).

 While other advocacy groups attributed their effective advocacy to the success gained in the past and the power of the people involved as explained by an advocacy coalition head,

 “*Our enabling factors were our past records, what we have been able to achieve in the past without any major support, I think they were actually moved by the fact that we were able to achieve a certain level without support so that was what motivated this our advocacy group, coupled with the caliber of individuals here” *(C2).

####  Ability to interact and generate evidence using victims

 At the subnational level, the CSOs are seen to actively engage with the people in the community. They go around interacting with those in the grassroots and generate convincing evidence. In some instances, the members of the community are recruited to present themselves to the policy makers to drive home their points. This the CSOs confirmed was useful for effective advocacy.

*“…we even brought some of these women who had lost their child or those who cannot afford to pay their medical bills to speak for themselves during our campaigns” *(C2).

 The advocates first understudy the policy making actors and then decide who to meet and when. The process of engagement could be a very tortious track as getting an appointment sometimes takes a long while,

*“Most times we need to come severally before we can meet with the policy makers as they are understandably very busy” *(N1).

####  Use of advocacy influencers

 Our findings revealed that most groups with successful outcomes made use of advocacy influencers, as a means to get to the policy makers,

 “*Yes. If its areas we want to go see the Igwe, we use the chairman, health committee. The person selected, must be an influential figure.” *(N3).

 They are known to intercede and hasten policy processes as stated by a national advocate,

*“Yes, it was because the first lady was there, and that was a very big driving force and based on that it has succeeded, and we also once in while have meetings where we invite the wives of the governors….” *(P5).

 Advocacy coalition groups that made use of strategies like engagement of the public and consensus building perceived it to be of little benefit to them as it was very difficult to hold down the politicians after the event.

 “*We called them to a workshop where the community members were present, and we present our evidence, and they are forced to make promises” *(G3).

###  Ideas

####  The identification of MNCH as a problem in Nigeria

 Our findings reveal that the ability of the advocates to convince the policy community by shaping the definition of the problem of MNCH and stating that most causes of MNCH morbidity and mortality are preventable was effective. All the participants viewed MCH as a very big problem in the country, with little progress on addressing this. As one respondent states:

*“It is a problem because adequate attention is not given to the health of the woman and the child. There are no adequate facilities. The maternal health is supposed to be free, but it is not, and you find out that many women patronize unqualified traditional birth attendants due to these reasons” *(C1).

 Yet another respondent said*, “I want to tell you one thing that Bill Gates said when they had Nigeria Business Round table, that Nigeria is the most dangerous country for a woman to deliver a child and that is just the situation, because if you look at the rate of maternal and infant mortality, you will now find out that this is true.” *(P5).

####  Proper framing of the MNCH problem

 Framing the issue and proper presentation of the magnitude of the problem of MNCH in Nigeria was a strategy used by all the groups. This was done through the media in some instances, where the journalists created scenarios and pictures that were quite empathic. The use of national figures of the death of mothers and children and comparing these with other low-income countries made it easier. One of the development partners said:

*“…we have relationship with the press and media which is very good, you can’t do advocacy without talking with the media, one of the good things is that we have very strong relationship with media in Nigeria and globally, so we use them whenever we want to advocate for faster results” *(D2).

 They also portray these as human rights and social justice issues, such that the citizens then demand for their rights during election campaigns. As a result of persistent publicity, MNCH issues became a significant topic during political campaigns. This is further highlighted by the fact that the women form a significant percentage of voters (30%) and the MNCH issue is guaranteed to attract their attention and votes.

 MNCH in Nigeria has been linked to many conferences attended by the country as a member states at which they signed memorandum of understanding, such as: Maputo protocol by decisions taken at the 1987 Nairobi conference on safe motherhood initiative; the 2000 Millennium summit that mapped out the 8 priority goals to be achieved by 2015, of which 4 and 5 targeted the reduction of maternal and child mortality respectively, at the end of which came the selection of SDGs and the development of a global strategy (Every woman every child) for Women’s, Children’s and Adolescents’ Health (2016-2030) by H6 agencies.

###  Issue characteristics

####  The presentation of MNCH problem

 The characteristics of the issue of MNCH has made it easier for advocacy to take place. First was the common nature of the problem and the fact that it affected a large number of the vulnerable population as stated by most respondents. Second was the availability of data from reliable sources showing the bad indices of MNCH in Nigeria Nigeria’s position was seen as worse than many low-income countries with less resources than Nigeria. The actors adopted greater use of quantifiable indicators, such as use of survey findings by international organizations like UNICEF that presented maternal mortality rates as higher (814/100 000) than the current rate being helped draw attention and resources to the issue when advocated for. All these led to the sustainability of issue attention on the country’s political agenda and gave a good understanding of the MCH problem, thus creating an enabling environment for the advocates. Third is the availability of resources for adequate intervention if the political will is present.

## Discussion

 This paper shows the role of advocacy in maintaining political commitment and attention to an issue over time. Studies have shown that MCH policy issues have been fluctuating in terms of commitments received from the government over time,^28,29^ coupled with the issue of falling donor commitment with little or no sustainability plans.^30^ This is consistent with Crichton’s analysis in 2008 where Joanne used the Grindle and Thomas framework to show the fluctuation in Kenyan government’s commitment to family planning policies.^31^ He concluded that despite the increase in policy space experienced, they did not believe that they had achieved success.^31^

 The competing health needs of diverse populations and ever reducing resources available to support these needs often serve as the drive for the initiation of advocacy to improve health outcomes. Various MNCH advocacies were carried out by actors who were motivated not just by a logic of consequences but by a logic of appropriateness.^32^ Some of these advocates in Nigeria formed coalitions that possess the characters identified internationally by authors as being an enabling factor for success such as gaining status, access, resources and diversity in groups.^32-34^ The presence of strong institutions and coordination of the actors involved in advocacy enabled the acquisition of political support. This contrasts with the case of the safe motherhood as shown by Shiffman and Smith where it was difficult to mobilize global support for maternal mortality reduction due to weak guiding institutions.^26^

 The study findings reveal that most advocates went through policy influencers. In Nigeria, the wives of the governor’s forum is a powerful group that was identified as influential and contributory to the achievement of advocacy goals. These findings are consistent with results from previous studies.^35,36^ Other policy influencers identified were celebrities who supported advocacy through fund-raising, film making, writing articles, meeting supporters, attending rallies, signing petitions or through donations. It can also involve less visible work behind the scenes like meeting policy makers or arranging such meetings between them and the organizations they support.^37,38^ They effectuate the goals of the advocates that engaged them as their messages have a wider reach because of their position and the number of followers they have on social media. This resonates well with Nigeria as the entertainment sector has grown very strong and is second only to Hollywood and Bollywood, and so explains the much attention received by these celebrities. There is need to identify more of them and engage them to use their personal and professional experiences to persuade policy change in MNCH as celebrity advocacy has been found to be very effective.^39^

 MNCH was aligned with global and national norms which are both strong and favourable. The advocacy actors framed the issue to align with the international priorities such that the problems were well portrayed and understood by all in the country. This is a strategy commonly used as shown in other studies.^8,40-43^ It is also called shift in understanding, which attracted the attention of the policy makers and politicians and guided their future actions in the adoption of Saving One Million Lives Program For Results (SOML PforR). These findings are consistent with other studies.^40-43^ Advocacy groups facilitated country policy adoption and the development of roadmaps for MNCH in African countries,^44^ of which Nigeria was one of those countries. This is worth emulating as it’s effectiveness in advocacy has been widely portrayed.

 The findings reveal that the advocates were able to show that the MNCH issue was severe but could be prevented if certain interventions are put in place as shown in other countries.^45-47^

 The use of media played a great role as observed by some authors that intense media attention increases the importance of public health issues.^48-50^ A political scientist, Bernard Cohen also noted that the media may not be successful most of the time in telling people what to think but it succeeds in telling them what to think about and how to think about a story,^51^ while other authors states that mass media creates our picture of the world.^52^ It has been shown that when the media chooses and concentrates on an issue, it makes the issue look more important than the others. Media advocacy is an important method of building public and governmental commitment to MNCH as stated by a Nigerian author.^53^ This is because in Nigeria, everyone is tuned to one form of media or the other, even in the rural areas with no electricity, batteries are used to power devices. Engaging with the media to improve accurate reportage and coverage of MNCH problems in Nigeria is critical to framing the agenda and holding policymakers accountable to addressing these critical issues as it has been found to be faster, has more widespread, and it’s more effective.

 The barriers to effective advocacy identified in this study resonates with problems identified by other authors as constraining advocacy in their countries.^50-54^ The issue of top-down approach to policy formulation is a common phenomenon in Nigeria because of the wide nature of the country in terms of the population, culture, religion and beliefs. Making decisions that will improve the health of over 196 million citizens is not easy and changing people’s conditions is an inherently political process that demands decision makers struggle with competing interests for inevitably limited resources. They therefore need sustained advocacy for formulation of policies that will benefit all groups and for the implementation of policies adopted at the State levels. This is because of the decentralized nature of the country, where states are not mandated to carry out federal policies.

 The limitation of this study is that it is difficult to attribute some of the outcomes stated in this paper solely to the gains of advocacy. In policy settings, this is almost impossible since randomizing advocacy interventions in a porous and well-connected policy field is infeasible.^55^ Through tracking and triangulation of the advocacy activities with interviews to support the policy landscape, we were able to highlight some of the contributions of advocacy. Our recruitment strategy may have an influence on how generalizable the findings are to the wider population. The recruitment procedure may have resulted in a larger proportion of stakeholders who were well informed. However, the framework used, the themes, conclusions and recommendations presented are useful and applicable to other contexts.

###  Implication for practice

 This study builds upon already existing policy analysis framework to examine contributions of policy advocacy interventions to national and sub-national MCH policy sphere. The Shiffman and Smith framework on factors influencing political priority for global health issues has identified issues to be considered for practice such as actor power which in this case varied, but most impact was made by the groups that had influential members in their coalition. The need for identified issues to be framed to resonate with global agenda. Also, the use of media for wide publicity is very useful while advocating. Identifying the right political context which in this case characterized by the election period and the change in government during which the issue was highlighted by the advocates with the use of numerical indicators to show the worsening maternal and child mortality rates which could be changed with the use of easily implemented interventions as shown in other countries as well.^26,42,43^ The identification of barriers to advocacy was necessary to highlight the issues that need to be addressed in order to achieve advocacy goals.

## Conclusion

 In conclusion, this paper generated insight to advocacy in MCH policy issues through the application of an agenda-setting framework to examine contributions of policy advocacy activities at the national and sub-national level.^26^ It hopes to advance the field of health policy analysis in low- and middle-income countries by showing how the use of framework offers a useful approach for organizing and analyzing data. It identifies effective strategies used by advocates to increase national and State attention for MCH issues in Nigeria and recommend their use in identifying legitimate problems, such as formation of advocacy coalition groups; framing MCH issue and linking it to the larger agendas of SDGs post unachieved MDGs, and to women’s and child’s right; taking advantage of national level policy windows related to change in government and political parties. Our study provides insights into issues that groups advocating for health issues at the national level will need to consider in strengthening the results of advocacy.

## Acknowledgments

 We are grateful to the Health Policy Analysis Fellows Mentorship Programme mentors, the Institute of Public Health, University of Nigeria and the Health Policy research group, who provided an institutional support to complete this study. We are also very grateful to the study participants.

## Competing Interests

 The authors declare that the research was conducted in the absence of any commercial or financial relationships that could be construed as a potential conflict of interest, or of competing interests.

## Data Availability Statement

 The dataset for this study is available and can be released on request.

## Ethical Approval

 This study was carried out in accordance with the recommendations of the guidelines of the University of Nigeria research policy and the University of Nigeria Senate Research Grants Committee. The protocol was approved by the University of Nigeria teaching Hospital Ethics Committee. All participants gave written informed consent in accordance with the Declaration of Helsinki.

## Funding

 Financial support for this study was provided by a grant from the Alliance for Health Policy and Systems Research, Health Policy Analysis Fellows Mentorship Programme under the World Health Organisation.

## References

[1] Black RE, Levin C, Walker N, Chou D, Liu L, Temmerman M (2016). Reproductive, maternal, newborn, and child health: key messages from Disease Control Priorities 3rd edition. Lancet.

[2] Walker N, Yenokyan G, Friberg IK, Bryce J (2013). Patterns in coverage of maternal, newborn, and child health interventions: projections of neonatal and under-5 mortality to 2035. Lancet.

[3] Bryce J, Arnold F, Blanc A, Hancioglu A, Newby H, Requejo J (2013). Measuring coverage in MNCH: new findings, new strategies, and recommendations for action. PLoS Med.

[4] Lassi ZS, Majeed A, Rashid S, Yakoob MY, Bhutta ZA (2013). The interconnections between maternal and newborn health--evidence and implications for policy. J Matern Fetal Neonatal Med.

[5] Buse K, Mays N, Walt G. Making Healthy Policy. London, UK: McGraw-Hill Education; 2012.

[6] Carpenter RC (2007). Setting the advocacy agenda: theorizing issue emergence and nonemergence in transnational advocacy networks. Int Stud Q.

[7] Nadelmann EA (1990). Global prohibition regimes: the evolution of norms in international society. Int Organ.

[8] Finnemore M, Sikkink K (1998). International norm dynamics and political change. Int Organ.

[9] Goetz AM, Gaventa J. From Consultation to Influence: Bringing Citizen Voice and Client Focus into Service Industry. IDS Working Paper 138. Brighton, Sussex: Institute of Development Studies (IDS); 2001.

[10] Moran M, Rein M, Goodin RE. The Oxford Handbook of Public Policy. Oxford University Press; 2006.

[11] Nelson BJ. Making an Issue of Child Abuse: Political Agenda Setting for Social Problems. Chicago, IL: University of Chicago Press; 1986.

[12] Schmidt M, Joosen I, Kunst AE, Klazinga NS, Stronks K (2010). Generating political priority to tackle health disparities: a case study in the Dutch city of The Hague. Am J Public Health.

[13] Gilson L, Orgill M, Shroff ZC. A Health Policy Analysis Reader: The Politics of Policy Change in Low- and Middle-Income Countries. Geneva: World Health Organization; 2018.

[14] Reich MR (1995). The politics of agenda setting in international health: child health versus adult health in developing countries. J Int Dev.

[15] Christoffel KK (2000). Public health advocacy: process and product. Am J Public Health.

[16] Clavier C, de Leeuw E. Health Promotion and the Policy Process. United Kingdom: Oxford University Press; 2013.

[17] Shiffman J (2007). Generating political priority for maternal mortality reduction in 5 developing countries. Am J Public Health.

[18] Okonofua F, Lambo E, Okeibunor J, Agholor K (2011). Advocacy for free maternal and child health care in Nigeria--results and outcomes. Health Policy.

[19] World Health Organization (WHO). Advocacy Strategies for Health and Development: Development Communication in Action. Geneva: WHO; 1995. Available from: https://apps.who.int/iris/handle/10665/70051.

[20] Tsui J, Hearn S, Young J. Monitoring and Evaluation of Policy Influence and Advocacy. ODI Working Paper Series. Vol 395. London: ODI; 2013..

[21] Sabatier PA, Jenkins-Smith HC. The advocacy coalition framework: an assessment. In: Sabatier PA, ed. Theories of the Policy Process. Boulder, CO: Westview Press; 1999. p. 117166.

[22] Barasa EW, Cleary S, English M, Molyneux S (2016). The influence of power and actor relations on priority setting and resource allocation practices at the hospital level in Kenya: a case study. BMC Health Serv Res.

[23] Yin RK. Case Study Research: Design and Methods. 2nd ed. Thousand Oaks, CA: SAGE Publications; 1994.

[24] Creswell JW. Qualitative Inquiry and Research Design: Choosing Among Five Approaches. SAGE Publications; 2012.

[25] Tong A, Sainsbury P, Craig J (2007). Consolidated criteria for reporting qualitative research (COREQ): a 32-item checklist for interviews and focus groups. Int J Qual Health Care.

[26] Shiffman J, Smith S (2007). Generation of political priority for global health initiatives: a framework and case study of maternal mortality. Lancet.

[27] Walt G, Gilson L (2014). Can frameworks inform knowledge about health policy processes? Reviewing health policy papers on agenda setting and testing them against a specific priority-setting framework. Health Policy Plan.

[28] Mafiana JJ, Shen X, Hu W, Wang X (2022). Insight into Nigeria’s progress towards the universal coverage of reproductive, maternal, newborn and child health services: a secondary data analysis. BMJ Open.

[29] Uneke CJ, Sombie I, Keita N, Lokossou V, Johnson E, Ongolo-Zogo P (2017). Improving maternal and child health policymaking processes in Nigeria: an assessment of policymakers’ needs, barriers and facilitators of evidence-informed policymaking. Health Res Policy Syst.

[30] The World Bank. New Health Care Model Launches in Nigeria to Improve Women and Children’s Health. September 23, 2016. Available from: https://www.worldbank.org/en/news/feature/2016/09/23/new-health-care-model-launches-in-nigeria-to-improve-women-and-childrens-health. Accessed November 4, 2022.

[31] Crichton J (2008). Changing fortunes: analysis of fluctuating policy space for family planning in Kenya. Health Policy Plan.

[32] Olsen GR (2001). European public opinion and aid to Africa: is there a link?. J Mod Afr Stud.

[33] Shiffman J (2016). Network advocacy and the emergence of global attention to newborn survival. Health Policy Plan.

[34] Hong L, Page SE (2004). Groups of diverse problem solvers can outperform groups of high-ability problem solvers. Proc Natl Acad Sci U S A.

[35] Page SE. The Difference: How the Power of Diversity Creates Better Groups, Firms, Schools, and Societies. Princeton, NJ: Princeton University Press; 2007. Available form: https://hbr.org/2007/03/the-difference-by-scott-page. Accessed June 15, 2022.

[36] Mirzoev T, Green A, Nancy G, Stephen P, Philippa B, Ha BT (2013). Role of evidence in maternal health policy processes in Vietnam, India and China: findings from the HEPVIC project. Evid Policy.

[37] Chouliaraki L (2012). The theatricality of humanitarianism: a critique of celebrity advocacy. Commun Crit Cult Stud.

[38] Couldry N, Markham T (2007). Celebrity culture and public connection: bridge or chasm?. Int J Cult Stud.

[39] Brockington D, Henson S (2015). Signifying the public: celebrity advocacy and post-democratic politics. Int J Cult Stud.

[40] McInnes C, Kamradt-Scott A, Lee K, Reubi D, Roemer-Mahler A, Rushton S (2012). Framing global health: the governance challenge. Glob Public Health.

[41] Reubi D (2012). Making a human right to tobacco control: expert and advocacy networks, framing and the right to health. Glob Public Health.

[42] de Bernis L, Wolman Y. UNFPA Maternal and Newborn Health National Plans (Road Map) Assessment: African MNH Road Maps Assessment Report. New York: United Nations Population Fund (UNFPA); 2009. p. 58.

[43] Smith SL, Rodriguez MA (2016). Agenda setting for maternal survival: the power of global health networks and norms. Health Policy Plan.

[44] African Population and Health Research Center (APHRC). Maternal Health Task Force. 2016. Available from: https://www.mhtf.org/organization/african-population-and-health-research-center. Accessed January 5, 2023.

[45] Shiffman J, Smith S (2007). Generation of political priority for global health initiatives: a framework and case study of maternal mortality. Lancet.

[46] Keck ME, Sikkink K. Activists Beyond Borders: Advocacy Networks in International Politics. Ithaca, NY: Cornell University Press; 1998.

[47] Stone DA (1989). Causal stories and the formation of policy agendas. Polit Sci Q.

[48] Wallack L, Dorfman L. Putting policy into health communication: the role of media advocacy. In: Rice RE, Atkin CK, eds. Public Communication Campaigns. 4th ed. Thousand Oaks, CA: SAGE Publications; 2012. p. 337-50.

[49] Dorfman L, Krasnow ID (2014). Public health and media advocacy. Annu Rev Public Health.

[50] Planned Parenthood Federation of America. “Alarmed and Saddened” by Komen Foundation Succumbing to Political Pressure, Planned Parenthood Launches Fund for Breast Cancer Services. January 31, 2012. http://www.plannedparenthood.org/about-us/newsroom/press-releases/alarmed-saddened-komen-foundation-succumbing-political-pressure-planned-parenthood-launches-fun-38629.htm. Accessed January 10, 2023.

[51] Cohen SA (1987). The safe motherhood conference. Int Fam Plan Perspect.

[52] McDougall L (2016). Discourse, ideas and power in global health policy networks: political attention for maternal and child health in the millennium development goal era. Global Health.

[53] Odorume A (2015). Mass media health communication: imperative for sustainable health development in Nigeria. J Afr Stud.

[54] Christoffel KK (2000). Public health advocacy: process and product. Am J Public Health.

[55] Harris J, Nguyen PH, To Q, Frongillo EA, Menon P (2016). Progress in improving provincial plans for nutrition through targeted technical assistance and local advocacy in Vietnam. Health Policy Plan.

